# Construction of multi-component finite element model to predict biomechanical behaviour of breasts during running and quantification of the stiffness impact of internal structure

**DOI:** 10.1007/s10237-024-01862-2

**Published:** 2024-05-28

**Authors:** Jiazhen Chen, Yue Sun, Qilong Liu, Joanne Yip, Kit-lun Yick

**Affiliations:** 1 School of Fashion and Textiles, The Hong Kong Polytechnic University, Hung Hom, Kowloon, Hong Kong; 2 School of Fashion Design and Engineering, Zhejiang Sci-Tech University, Hangzhou, 310018 China

**Keywords:** Multi-component model, Finite element analysis, Breasts tissue, Stiffness, Running, Biomechanical analysis

## Abstract

This study aims to investigate the biomechanical behaviour and the stiffness impact of the breast internal components during running. To achieve this, a novel nonlinear multi-component dynamic finite element method (FEM) has been established, which uses experimental data obtained via 4D scanning technology and a motion capture system. The data are used to construct a geometric model that comprises the rigid body, layers of soft tissues, skin, pectoralis major muscle, fat, ligaments and glandular tissues. The traditional point-to-point method has a relative mean absolute error of less than 7.92% while the latest surface-to-surface method has an average Euclidean distance (*d*) of 7.05 mm, validating the simulated results. After simulating the motion of the different components of the breasts, the displacement analysis confirms that when the motion reaches the moment of largest displacement, the displacement of the breast components is proportional to their distance from the chest wall. A biomechanical analysis indicates that the stress sustained by the breast components in ascending order is the glandular tissues, pectoralis major muscle, adipose tissues, and ligaments. The ligaments provide the primary support during motion, followed by the pectoralis major muscle. In addition, specific stress points of the breast components are identified. The stiffness impact experiment indicates that compared with ligaments, the change of glandular tissue stiffness had a slightly more obvious effect on the breast surface. The findings serve as a valuable reference for the medical field and sports bra industry to enhance breast protection during motion.

## Introduction

**Fig. 1:**
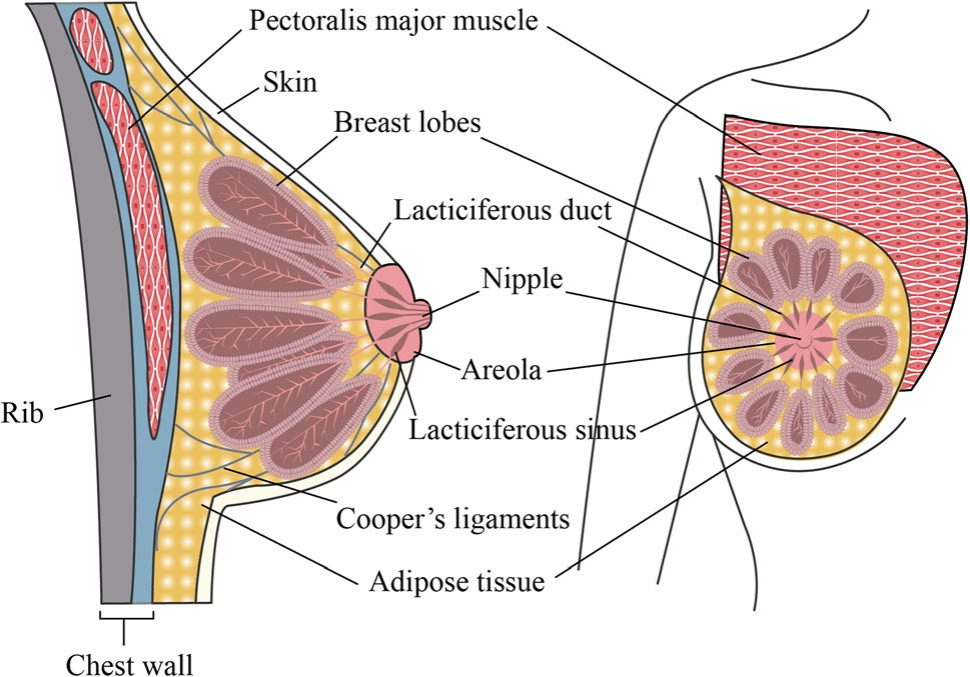
Breast anatomy

Nowadays, women are increasingly focusing on their breast health. Over the years, work has been done to advance studies on the anatomical structure of the breasts, supported by quantitative imaging data and anatomical evidence (Gaskin et al. 2020a, 2020b; Rehnke et al. 2018). The breasts are attached to the chest wall by a series of complex anatomic structures (McGhee and Steele 2020). The primary components of the breasts include the skin of the breasts and internal components, which include the adipose (collection of fat cells) and glandular (milk production) tissues, fascia muscles, Cooper's ligaments, blood vessels, and retromammary space. The glandular tissues comprise 15–20 glands that are known as lobes, which are found in the fibrous and adipose tissues, and a network of ducts that are extended towards the nipples (Darlington 2022). The Cooper's ligaments are connective tissues that provide the primary anatomical support to the breasts by supporting the fibrous and adipose tissues (McGhee et al. 2007, 2013; Scurr et al. 2009, 2010). Gaskin et al. (Gaskin et al. 2020b) stated that the Cooper's ligaments form a 3D mesh structure that includes the fat and mammary lobules. During motion, these ligaments can affect the shape and mechanical behaviour of the breasts in the upright posture (Gefen and Dilmoney 2007). Figure 1 shows the anatomy of the breast.

All of the components of the breasts are soft, and there is no skeleton frame (Ramião et al. 2016), thus the breasts have little anatomical support except for the Cooper’s ligaments. During sports participation, excessive movement can lead to significant displacement of the breasts, which results in breast pain (Green et al. 2003; Page and Steele 1999). The source of this pain is challenging to observe objectively, but often characterised by discomfort, sagging, and excessive movement during physical activities (Page and Steele 1999). Nevertheless, the scientific literature on the relationship between breast anatomy and breast movement is scarce. However, research studies on the breast structure and movement could greatly contribute to understanding issues related to the breasts, such as diagnosing and treating pathologies, reconstructing the breasts post-mastectomy, caring for the breasts post-cosmetic surgery, and designing supportive sports bras (McGhee and Steele 2020).

In terms of breast kinematics, researchers have quantified breast displacement and acceleration by using infrared cameras or motion capture systems with markers (Lee et al. 2021; Lu et al. 2016; Mills et al. 2016; White et al. 2015, 2009). Typically, they can only capture motion data from a limited amount of surface markers, such as the nipples markers. However, given the soft tissues of the breasts (Ramião et al. 2016) and their unique rheological properties and biomechanical behaviours (Gefen and Dilmoney 2007; Haake and Scurr 2010, 2011; Sun et al. 2019a, b), using a limited number of markers to examine the complex movements of the breasts might result in considerable information loss and an incomplete description of the breast motion. The latest 4D scanning technology can acquire dynamic surface data during motion, which can help to make up for the above defects in breast kinematics research (Pei et al. 2020, 2021).

Recently, the finite element method (FEM), which is extensively used in biomechanical research, has become a critical research approach for studying breast motion through dynamic FE modelling. Typically, 3D scanning technology captures the 3D point clouds of the surface of the body which are used to create the geometrical and dynamic FE models of the breasts. With the use of reflective markers, researchers can successfully acquire the motion trajectories of breast motion by using infrared cameras or motion capture systems. The motion trajectory data can then be compared with FE simulation results to verify the accuracy of the FE model. In research on the biomechanics of the breasts during running, Chen et al. (2013) utilised a 3D dynamic FE model to simulate nonlinear breast motion. Their breast model comprised the torso, breast, and three layers of skin. Cai et al. (2018) developed a theoretical mathematical model to study the viscoelastic behaviour of the breast during free vibration and motion. A 3D FE model that included the torso and breast to predict breast motion was established. Liang et al. (2019) conducted an FE analysis to simulate displacement under static and dynamic contact conditions by using a model that comprises a rigid torso, breasts, and the subcutaneous tissues. Similarly, Zhang et al. (2022) used a nonlinear biomechanical FE model to simulate breast deformation during arm abduction among elderly women. Their model included the torso, breasts, pectoralis major muscle, and bones. These studies have given us a better understanding of breast movement, but there are several limitations. Firstly, traditional 3D scanning systems can only show surfaces during controlled static postures and some limited static postures, thus failing to capture dynamic data as a reference for FE models. The Vicon motion capture system can only obtain motion data for several key points, and cannot show surface changes (Lee et al. 2021; Lu et al. 2016; Mills et al. 2016; White et al. 2015, 2009). Secondly, these studies have mainly primarily concentrated on constructing dynamic models of the breasts and torso using a predefined set of material parameters. However, they have largely overlooked the biomechanical characteristics of the internal components of the breasts and the influence of their material properties (Sun et al. 2019a, b).

In the medical field, researchers have proposed static breast models based on the different components of the breasts by using magnetic resonance imaging (MRI). Sturgeon et al. (2016) used a template-based FE model of the breast which could be applied to its pre-segmented voxelised model. The FE model automatically adapts to the geometry of a given model of the breast and body, and the material properties of each element are set based on the segmented voxels contained within the element. Mira et al. (2018) developed and evaluated a biomechanical FE model of the breasts that consider the adipose tissues, muscles, skin, suspensory (Cooper’s) ligaments, and pectoral fascia muscles. The stress-free geometry of the breasts and constitutive models were constructed by using magnetic resonance images. However, the Cooper’s ligament and tissues of the model were described by using line units, which do not conform to the anatomical structure of the breasts. Additionally, the glandular tissues were neglected. Unfortunately, as MRI is done in the static state, these studies which used MRI have only focused on static analyses of the mechanical behaviour and neglected to simulate breast motion with large strains, which is a research gap. In addition, the medical field is also concerned about the effect of stiffness on breast surface movement. Because changes in the stiffness of breast tissue are an important indicator of breast abnormalities, including tumours or cancerous lesions (Boyd et al. 2014). Therefore, it is crucial to address these research gaps by constructing a multi-component nonlinear large-strain model to study breast motion and its applications (McGhee and Steele 2020; Sun et al. 2019a, b). The model can enhance the current understanding of the interaction among the breast components and the impact of external forces on these components, and can also explore the effect of material stiffness on the breast surface. The findings could act as a valuable reference for the diagnosis of non-invasive canceration in the medical field and the innovative development of protecting the breast during movement in the sports bra industry.

Given the limitations of previous studies, the main objective of this study is to construct a multi-component dynamic FE model of the breasts during running and further to explore the impact of internal components stiffnesses on breast surface. This study will employ the latest 4D scanning technology, which captures surface data including time axes for data input and dynamic model verification. The motion data primarily comprise motion displacement in two directions (mediolateral and vertical directions). Based on the latest research on breast anatomy, construction of a multi-component dynamic FE model of the breasts will be done to study the biomechanical behaviour of the components of the internal structure of the breasts. This model will enable to examine the impact of varying stiffness levels of internal components on breast surface.

## Experiments and methods

### Experiments

#### Subject

With age, the ability of the components of the breasts to provide biomechanical support to the breasts gradually declines (Coltman et al. 2017; Tonkelaar et al. 2004). To ensure the functional integrity of breast tissue, a healthy woman who is 27 years old is recruited for this study. Her height is 160 cm, and weight is 58 kg. Her bra size is 80C (metric bra sizing system). A 4D scanning system (3dMD system, US) was used to scan this subject, and build a geometric model of the body and breasts with static data and verify the subsequent FE model with dynamic data. The subject gave informed consent before participating in the experiment, which was approved by the Human Subjects Ethics Sub-committee of university of the first author (Approval No. HSEARS20151207004).

#### Data collection

**Fig. 2:**
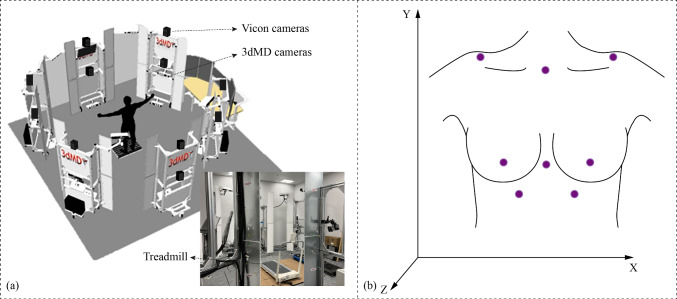
Experiment setting: **a** 4D scanning with 3dMD system and Vicon motion capture system, and **b** position of markers

The subject was scanned with the 3dMD system while she was running, which can capture 120 frames per second with 30 machine vision cameras (see Fig. 2a) and provides the 3D point clouds of the surface of the breasts at a high-frame rate (Pei et al. 2020, 2021). The 3D data of the breast morphology can be obtained with time, so that the dynamic changes and regional deformations of the surface of the breasts, stretching of the skin and geometric changes of the breasts can be analysed to provide clarity on the soft tissues and boundaries of the entire breast during movement. Biomechanical evaluations of breast motion during running were conducted using a motion capture system (VICON Vero, Nexus 2.15, UK), capturing 120 frames per second with 12 digital cameras (Fig. 2a).

The upper half of the body of the subject was required to be undressed to adhere eight flat markers with an outer diameter of 6 mm and a central hole diameter of 3 mm onto her (see Fig. 2b). When the scanning and capturing process began, she was in a standing position on the treadmill (XQIAO, China) for 5 s, with her arms slightly apart from her torso, as this is the standard posture for 3D scanning of the body (Choi and Ashdown 2011). Then the treadmill gradually started and accelerated to a set speed (6 km/h). Then the subject kept running for 10 s. As seen in Fig. 2b, the X and Y directions represent the mediolateral and vertical directions, respectively. The Z direction represents the anteroposterior direction.

### 3D image processing

The motion data obtained from the Vicon motion capture system was denoised by the low-pass filter (zero lag 4th order Butterworth, cut off frequency fc = 6 Hz) (Mansour et al. 2015; Winter et al. 1990) as FE dynamic model motion data input. The static 3D data from 4D scanning was imported into Geomagic Design X software (US) to generate the 3D surface of the body. The head and limbs were removed, holes were filled and the torso was smoothed. The upper body was segmented into a rigid torso (i.e., the rigid bone structure) and a layer of subcutaneous soft tissues according to the thickness (25 mm) of the soft tissues (Kim et al. 2012) of the chest wall (see Fig. 4). The breasts were extracted from the soft tissues.

### Gravity-free breast model construction

**Fig. 3:**
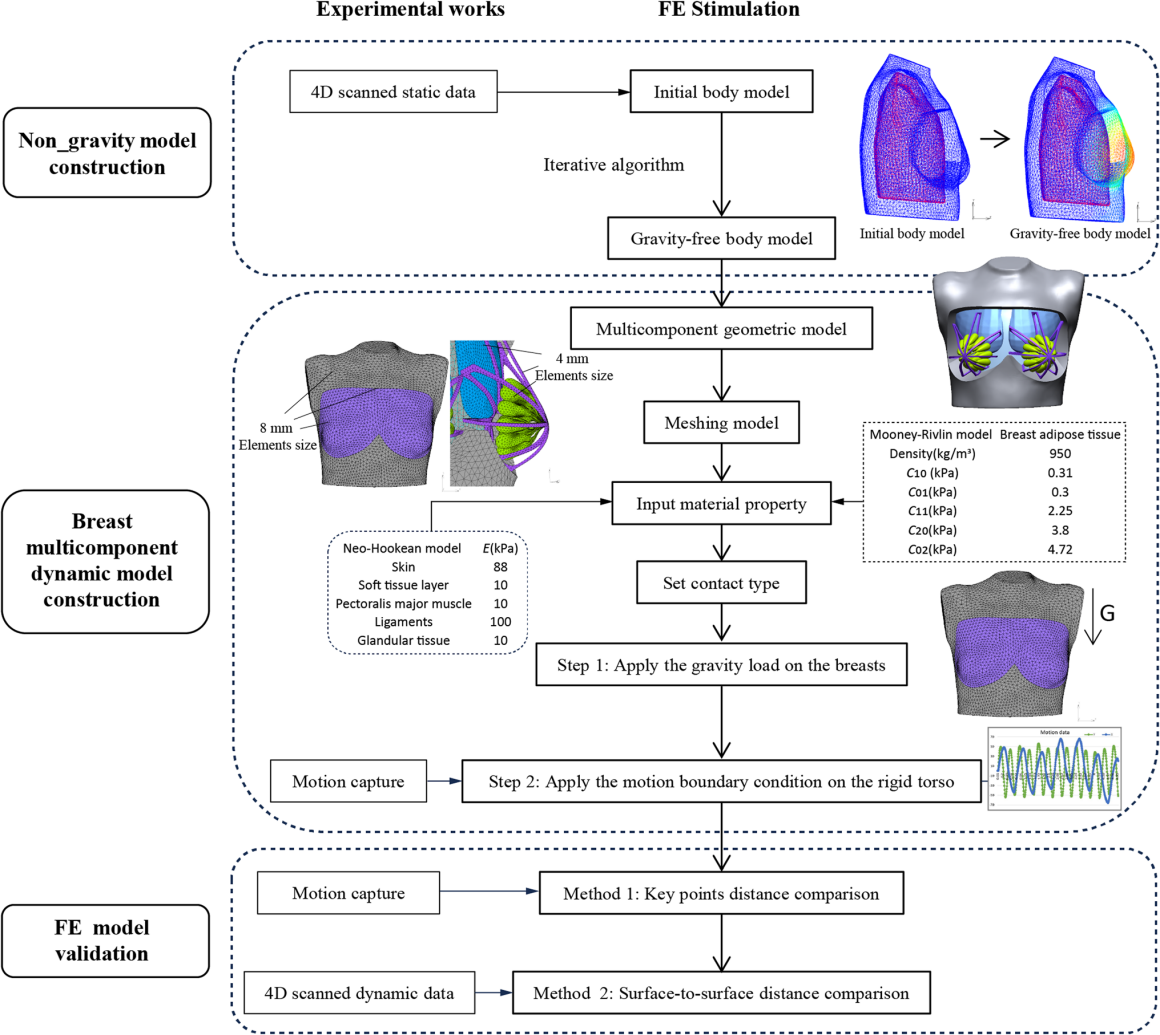
Research process of multi-component breast model construction

When 4D scanning the subject, her breasts were deformed by gravity. To simulate the deformation of her breasts during movement due to the effects of gravity, it is necessary to first obtain a gravity-free breast model as the initial geometric configuration before constructing a multi-component dynamic FE model of the breasts as shown in Fig. 3. The breasts and layer of subcutaneous soft tissues in the FE model were meshed using 8 mm tetrahedron elements in MSC Apex software (US) based on mesh convergence analysis. The material properties of the soft tissues were obtained by referring to Samani and Plewes (Samani and Plewes 2004). Then, the gravity-free breast model was calculated by using an inverse algorithm (Eder et al. 2014). The process involved applying upward gravity and then downward gravity onto the breasts. By comparing the shape of the breasts with downward gravity and that of the 4D scanned breast, the initial shape of the breast without gravity was iteratively obtained.

### Multi-component breast model construction

#### 3D geometry and mesh model

**Fig. 4:**
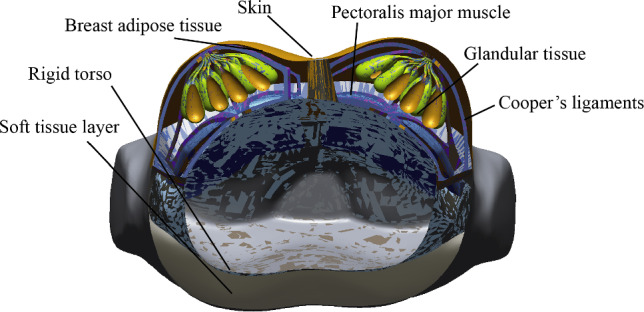
Multi-component geometric model

The glandular tissues have 15–20 lobes arranged radially around the nipple in the breast anatomy (see Fig. 1), and mostly in the centre of the breast (Hassiotou and Geddes 2013; Huang et al. 2011). These lobes, which have a cystic structure (Gefen and Dilmoney 2007; Gui et al. 2004; Hassiotou and Geddes 2013), include lobules and ducts, and are found in the fibrous and adipose tissues. A network of ducts that carry milk to the nipples (Darlington 2022; McGhee and Steele 2020). Therefore, in the geometric model of the mammary lobes (see Fig. 4), a stretched angular sphere (with a diameter of 15–18 mm) was considered to characterize the mammary lobules. The lower part of the lobule was connected with a thick to thin tubular duct (with a diameter of 2–4 mm) (Hassiotou and Geddes 2013). The duct curvature should match the curved surface of the nipple areola. 18 sub-models of the lobes were arranged into a double circular array according to the inner and outer layers. Gathering 18 ducts in a circular body near the nipple, the sub-model of the glandular tissues was constructed.

The model of the Cooper’s ligaments was simplified as a radial network with a 3D closed structure. The ligaments were attached forward to the circular body of the glandular model and backward to the pectoralis major muscle. The anterior and posterior ligaments conformed to the curvature of the breast skin surface and the pectoralis major surface, respectively. The upper two-thirds of the breast was attached to the pectoralis major muscle (Woodworth et al. 2017).

The pectoralis major muscle was placed along the surface of the chest wall of the rigid torso. The pectoralis major muscle was formed by stretching the sketch along the curvature path (chest wall) to create a solid shape. The pectoralis major contour was trimmed based on anatomy (Woodworth et al. 2017). The muscle exhibited a thicker middle section, approximately 12 mm on average (ranging from 10–15 mm), while the surrounding edges were less than 5 mm thick (Bueno et al. 2017; Ishii et al. 2018; Koo et al. 2010). The sub-models of the pectoralis major muscle, Cooper’s ligaments, and adipose tissues were built using a Boolean operation. The average thickness of healthy breast skin is about 1.51 mm (Huang et al. 2008). Thus, the skin of the entire body was configured as a 2D shell with a thickness of 1.5 mm.

Meshing is particularly important for this model because the geometric model is complicated and the difference in size between the sub-models is large. When meshing the sub-models, it is necessary to ensure that the contact surfaces of the sub-models are common nodes, in order to facilitate the calculation. All of the solid sub-models were meshed by using four node tetrahedrons and the breast skin were meshed by the triangular thin shell element in MSC Apex software (Yu et al. 2016). To prevent mesh penetration, as shown in Fig. 3, the pectoralis major muscle, Cooper’s ligaments and glandular tissues are divided by using the same small mesh size (4 mm). The layer of soft tissues and adipose tissues near the contact surfaces of ligaments and glandular tissues are meshed with 4 mm elements, while the remaining areas are meshed with 8 mm elements. The skin is meshed by using 8 mm elements. The number of solid elements is 77,446 for the layer of soft tissues, 202,809 for the adipose tissues, 34,041 for the pectoralis major muscle, 7253 for the Cooper’s ligaments and 174,848 for the glandular tissues. The number of shell elements is 8000 for the skin.

#### Material properties

In this study, all the soft tissues were considered as a homogenous isotropic hyper-elastic type of material with nonlinear behaviour. Due to the quasi-incompressibility of the soft tissues, the Poisson's ratio (*v*) of these components was set to 0.49 (Mira et al. 2018; Zhang et al. 2022). The strain energy density function *W* (Rivlin 1948) describes the energy per unit volume which can be written as:

1 $$ W(\overline{I}_{1} ,\overline{I}_{2} ) = \sum\limits_{i,j = 0}^{n} {C_{ij} (\overline{I}_{1} - 3)^{i} (\overline{I}_{2} - 3)^{j} } $$

where *C*_*ij*_ represents the hyper-elastic parameter that characterizes the nonlinear elastic behaviour of the soft tissues (Samani and Plewes 2004), $\overline{I}_{1}$ and $\overline{I}_{2}$ are the first and second invariants of the components of the left Cauchy-Green deformation tensor ${\overline{\mathbf{B}}}$ in the form of:

2 $$ \overline{I}_{1} = tr({\overline{\mathbf{B}}}) $$

3 $$ \overline{I}_{2} = \frac{1}{2}[(tr({\overline{\mathbf{B}}}))^{2} - tr({\overline{\mathbf{B}}}^{2} )] $$

where $tr$ means the trace of the matrix. ${\overline{\mathbf{B}}} = {\mathbf{F}} \cdot {\mathbf{F}}^{T}$, where ${\mathbf{F}}$ is a deformation gradient, and ${\mathbf{F}}^{T}$ is the transposition of ${\mathbf{F}}$.

Equation (1) can be simplified into a Neo-Hookean model (Sang et al. 2015). The Neo-Hookean constitutive model is used to describe the nonlinear elastic behaviour of the skin, layer of soft tissues, pectoralis major muscle, Cooper’s ligaments and glandular tissues. The strain energy density function *W* derived from Eq. (1) is:

4 $$ W = \frac{\mu }{2}(\overline{I}_{1} - 3) + \frac{1}{d}(J - 1)^{2} $$

where $J$ is the determinant of the deformation gradient ${\mathbf{F}}$. ${\upmu }$ and $d$ are the initial shear modulus and material incompressibility respectively, which are related to the Young's modulus (*E*) and Poisson's ratio (*v*) for small deformations, respectively:

5 $$ \mu = \frac{E}{2(1 + v)};d = \frac{6(1 - 2v)}{E} $$

The Young's modulus (*E*) was set to 500 kPa for the skin (Zhang et al. 2022), 10 kPa for the layer of soft tissues, 10 kPa for the pectoralis major muscle (Wenger et al. 2007), 100 kPa (Wenger et al. 2007) for the Cooper’s ligaments, and 10 kPa for the glandular tissues (Gefen and Dilmoney 2007; Samani et al. 2001; Sturgeon et al. 2016).

In Eq. (1), when *n* is equal to 2, the type of material of the adipose tissues is referred to as a fifth-order Mooney-Rivlin material with 5 coefficients (C_10_, C_01_, C_11_, C_20_, and C_02_), which are defined to represent their nonlinear behaviour with larger deformations. According to Samani and Plewes (2004), the material coefficients of the adipose tissues are C_10_ = 0.31 kPa, C_01_ = 0.3 kPa, C_11_ = 2.25 kPa, C_20_ = 3.8 kPa, and C_02_ = 4.72 kPa. The density was set to 950 kg/m^3^ (adipose tissues) (Rajagopal et al. 2008), 1040 kg/m^3^ (Cooper’s ligaments) (Rajagopal et al. 2008), 1070 kg/m^3^ (glandular tissues) (Vandeweyer and Hertens 2002), and 1050 kg/m^3^ (pectoralis major muscle and layer of soft tissues) (Rajagopal et al. 2008). These material coefficients are consistent with those in the literature which suggests that the stiffness of the normal glandular tissues is 1 to 6.7 times that of the adipose tissues (Azar et al. 2002; McKnight et al. 2002).

#### Boundary conditions

In order to reach higher computational efficiency, the simulation was performed with the upper half of the body due to its symmetry. The symmetrical boundary condition was set in the Marc software (MSC. Marc 2020, US), designating the sagittal plane as the symmetry plane. The model of the upper half of the body was composed of a rigid torso and six parts of the soft tissues which are deformable. The contact type between the torso and soft tissues of the breasts was set to glued interaction. To simulate the natural breast, it is necessary to apply a gravity load onto the breast. In Fig. 3, a downward gravity acceleration of 9800 mm/s^2^ was exerted to the nodes of the breast model, including the adipose and glandular tissues and the Cooper’s ligaments. The motion data of the clavicle point, obtained from the Vicon motion capture system, were considered to be the boundary conditions for the motion displacement of the rigid torso in the two main directions of X and Y at 3 s.

#### Dynamic simulation

In Marc software, the static structure analysis was utilised to stimulate the process after applying gravity loading. Second, multi-component breast dynamic simulation has employed transient dynamic analysis, using the implicit dynamic method. The Full Newton–Raphson method was employed as an Iterative solver. The fixed time was 3 s and the constant time step was 0.005 s, with 600 steps.

### Validation method

Previous studies (Eder et al. 2014; Liang et al. 2019; Zhang et al. 2022) have compared FE simulated and experimental data results by using several key points, such as the clavicle, shoulder, and nipple points. The criterion for this difference is the relative mean absolute error (RMAE) (Phellan et al. 2021):

6 $$ RMAE = \frac{1}{N}\sum\limits_{i = 1}^{n} {\left| {\frac{{D_{EXP} - D_{FEM} }}{{D_{EXP} }}} \right|} $$

where $D_{EXP}$ and $D_{FEM}$ are the displacement of the key points obtained from the experiment and simulation, respectively. *N* is the number of sample data points.

Besides, the surface deviation needs to be considered. Using Python 3.9.16, the surface deviation was determined by the difference between the FE simulated deformed surface and 4D scanned data of *n* frames of a motion cycle. The surface-to-surface distance evaluation is defined as:

7 $$ d = \frac{1}{{N_{frame} N_{node} }}\sum\limits_{i = 1}^{{N_{frame} }} {\sum\limits_{j = 1}^{{N_{node} }} {\left\| {n_{j}^{(i)} - m_{j}^{(i)} } \right\|^{2} } } $$

where i,j represent the frame and node, respectively; $N_{frame}$ is the total number of frames; $N_{node}$ is the total number of nodes; and d is the average Euclidean distance between the FE simulated nodes $n_{j}^{(i)}$ and their nearest point $m_{j}^{(i)}$ from the 4D scanned mesh. The nearest point $m_{j}^{(i)}$ from the corresponding frame of the 4D scanned mesh was obtained by using the nearest point-to-plane.

## Results and discussion

### Validation results

#### Point-to-point evaluation

A point-to-point comparison was conducted by using the RMAE. For the clavicle and nipples, the RMAEs of the motion trajectory from the raw experimental data and FEM simulated results in the X and Y directions are obtained as shown in Table 1.

**Table 1:** RMAEs of key points

Key points	X	Y
Clavicle	4.82%	2.62%
Left nipple	7.92%	6.74%

The RMAE is the relative mean absolute error between the experimental raw data and FEM simulated result. The RMAE of the clavicle in the X and Y directions is 4.82 and 2.62%. The RMAE of the left nipple in the X and Y directions is 7.92 and 6.74%, respectively. RMAEs less than 7.92% are acceptable, so this FE model is valid under a point-to-point evaluation.

#### Surface-to-surface evaluation

**Fig. 5:**
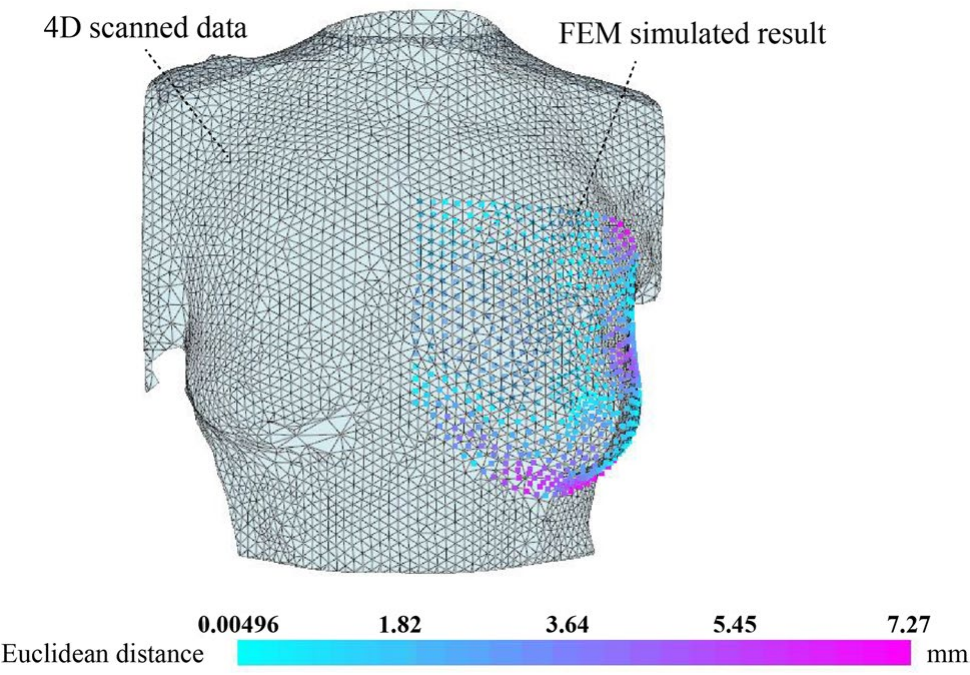
Surface-to-surface distance

The surface-to-surface distance evaluation *d* is shown in Fig. 5. The surface-to-surface distance evaluation method measures the difference between the surfaces based on 4D scanned data and the FEM simulated result, which contains relatively complete surface information. Figure 5 shows that the surface-to-surface distance *d* is 7.05 mm, which is acceptable (Eder et al. 2014; Mira et al. 2018). Therefore, this FE model is valid for a surface-to-surface evaluation.

### Displacements and acceleration analysis of different components of breast

**Fig. 6:**
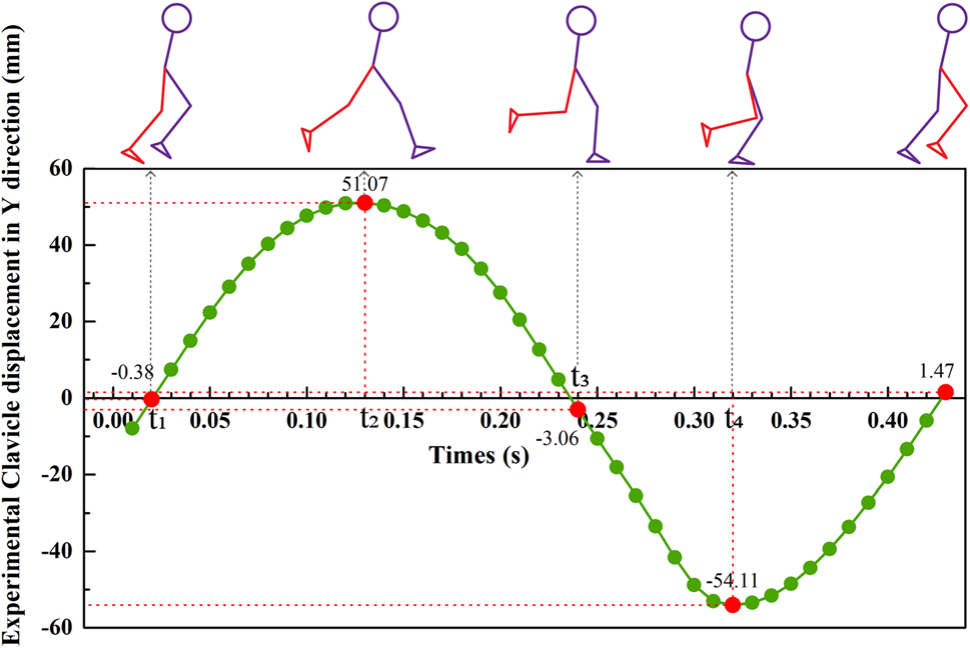
The experimental clavicle displacement in Y direction of half a gait cycle

Figure 6 shows the experimental clavicle displacement in Y direction from Vicon motion capture system. Half of the gait cycle has been defined by (Antonie et al. 2012). t_1_ is the moment when one foot has just left the floor, and t_3_ is when the foot makes initial contact with the floor. t_2_ and t_4_ are the highest and lowest displacement moments of the clavicle in the vertical direction, respectively.

**Fig. 7:**
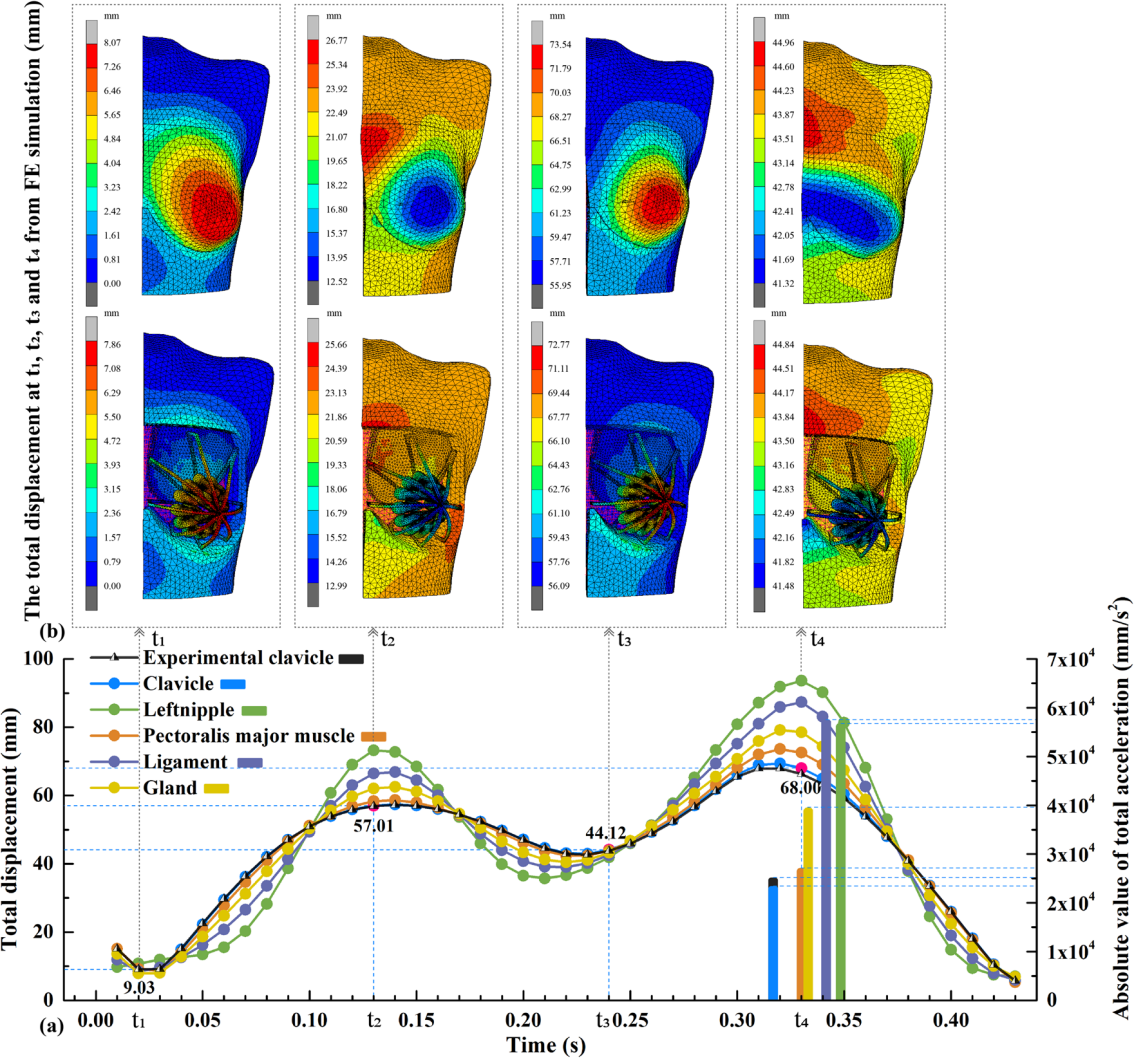
Total displacement and maximum acceleration: **a** experimental clavicle and simulated different components of breasts, and **b** Total displacement nephogram at t_1_, t_2_, t_3_, and t_4_

Figure 7 shows the total displacement and maximum acceleration of the different components of the breast from FE simulation. The total displacement is the vector sum of the displacements in three directions.

In half a motion cycle, the first peak is t_2_ (0.13 s). The displacement of the left nipple (73.24 mm) is the highest, while the clavicle shows the least displacement (57.01 mm). The second peak is t_4_ (0.32 s). Similarly, the highest displacement is also the left nipple (93.56 mm), while the clavicle shows the least displacement (68.00 mm). At maximum displacement, the following order of the displacement is observed: left nipple > Cooper’s ligament > glandular tissues > pectoralis major muscle > clavicle. This is related to the internal structure of the breast, which is proportional to the distance from the rigid chest wall. That is, the breast component that is located farther away from the chest will have a higher total displacement. When the motion reaches t_2_, the body is at the highest point in the Y direction of the running cycle. When the body approaches t_4_, the contact of the toe with the ground produces a ground reaction force, which results in the negative direction (braking) and relatively large acceleration. The breast components reach the maximum acceleration according to the following order: a__max_^ligaments^ (57.02 m/s^2^) > a__max_^left nipple^ (56.21 m/s^2^) > a__max_^glandular tissues^ (38.89 m/s^2^) > a__max_^pectoralis major muscle^ (26.55 m/s^2^) > a__max_^clavicle^ (22.80 m/s^2^). The slight difference in results between displacement is due to the ligament experiencing slightly higher acceleration than the nipple. This is because, at the moment of maximum impact, the Cooper ligament undergoes greater inertial force and deformation compared to the nipple. This is also one of the reasons why ligaments play a major supportive role and are susceptible to injury (Gefen and Dilmoney 2007).

### Biomechanical analysis of different components of breast

#### Biomechanical analysis of Cooper’s ligaments

**Fig. 8:**
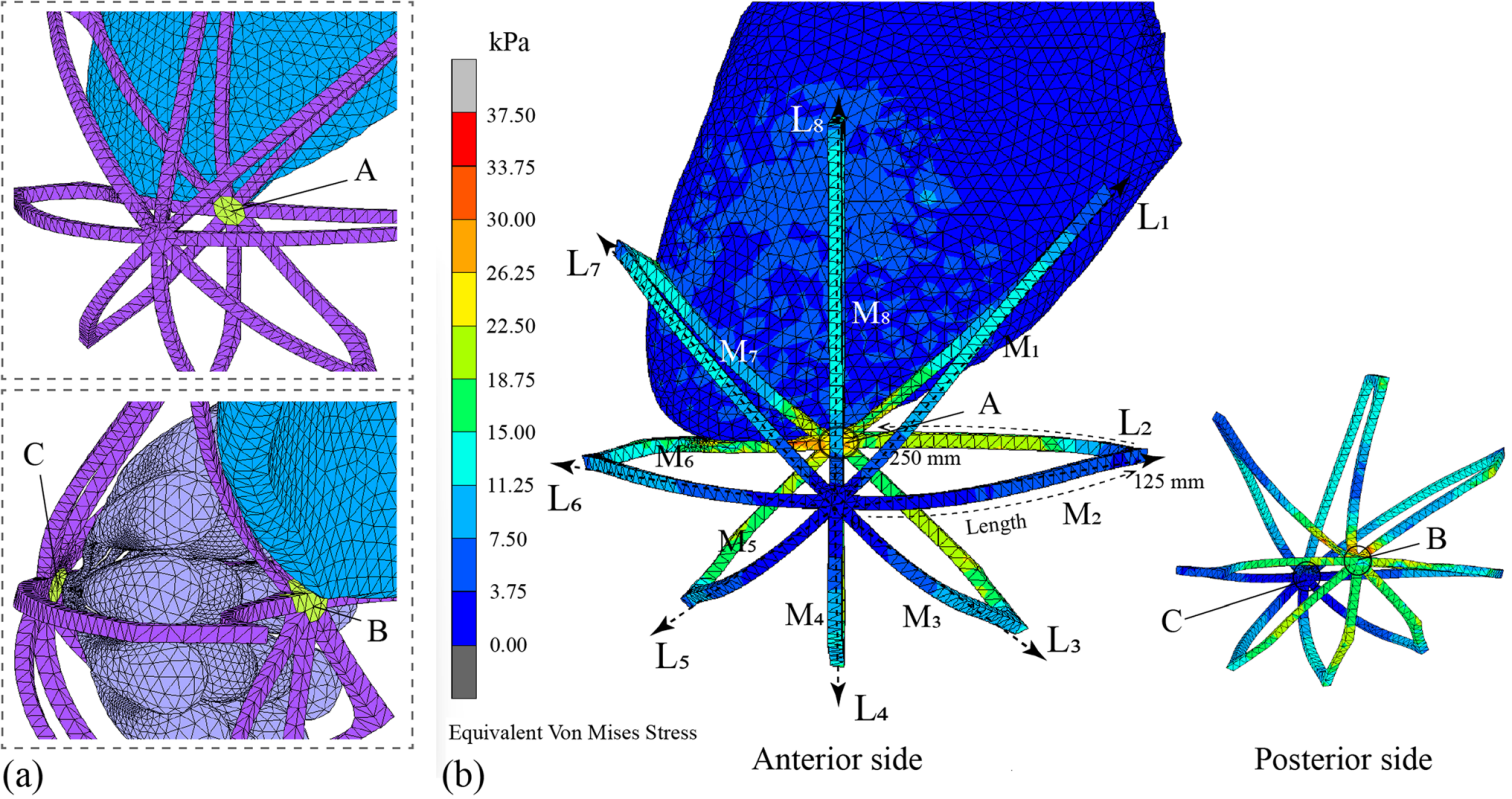
Equivalent von Mises stress of ligaments: **a** positions of three concerned areas, and **b** stress nephogram

Areas A, B, and C in Fig. 8a show the three different areas of the Cooper’s ligaments. A shows the area that is in contact with the adipose tissues behind the Cooper’s ligaments. B is the central area behind the Cooper’s ligaments which is attached to the pectoralis major muscle. Finally, C is the area of the Cooper’s ligament that is in contact with the glandular tissues. Figure 8b shows the equivalent von Mises stress nephogram at t_4_. The Cooper’s ligaments are described in eight directions: L_1_–L_8_.

**Fig. 9:**
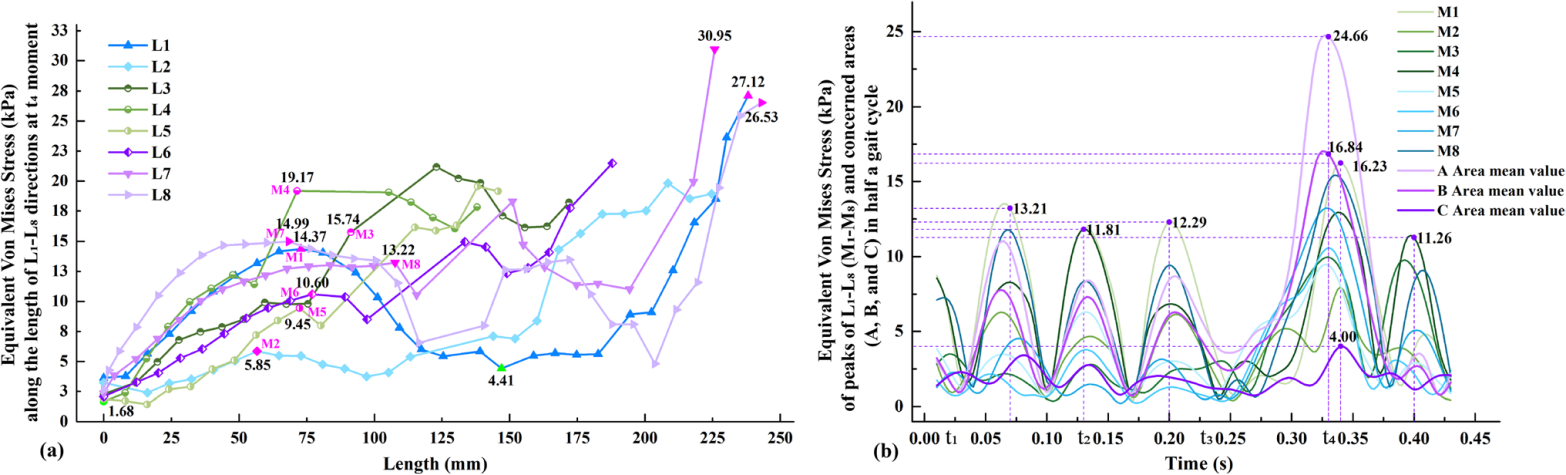
Equivalent von Mises stress of Cooper’s ligaments: **a** L_1_-L_8_ directions at t_4_ moment and **b** peaks of L_1_-L_8_ (M_1_-M_8_) and three concerned areas (A, B, and C) in half a gait cycle

Figure 9a shows the equivalent Von Mises stress along the length of the Cooper’s ligaments at t_4_ moment in the L_1_–L_8_ directions. t_4_ is the moment of the maximum amplitude. The plotted 0–125 mm length of the Cooper’s ligaments approximately describes their anterior side. The equivalent von Mises stress of the anterior ligaments ranges from 1.68 to 19.17 kPa. The maximum values in each direction, indicated by the pink highlights (M_1_–M_8_), are recorded. The length of the curve from 125–250 mm approximately represents the posterior side of the Cooper’s ligaments, which are attached to the pectoralis major muscle, as shown in Fig. 8b. In Fig. 9a, the equivalent von Mises stress of the posterior side of the Cooper’s ligaments varies from 4.41 to 30.95 kPa, which is slightly higher than that of the anterior side of the Cooper’s ligaments. It can be observed that within a length of 200–250 mm, the stress borne by the Cooper’s ligaments increases with more distance, and peaks to about 30 kPa. This suggests that the posterior side of the Cooper’s ligaments bears more stress than the anterior side of the Cooper’s ligaments. The equivalent von Mises stress in the L_1_, L_7_ and L_8_ directions is generally higher than in the other directions, which shows that the ligaments in these directions bears more stress and provide more support to the breasts during running.

Figure 9b shows the equivalent Von Mises stress of the M_1_–M_8_ nodes in half a gait cycle. Three stress peaks are found, that is, 13.21, 11.81, and 12.29 kPa, respectively. At t_4_, each node reaches its peak stress value. t_4_ coincides with the moment of maximum acceleration and therefore, yields the maximum stress. In terms of the three areas of concern, the mean values of the equivalent von Mises stress for A, B, and C are calculated respectively (see Fig. 9b). Area A is subjected to the highest stress, and peaks at 24.66 kPa, followed by B, which peaks at 16.84 kPa. These findings suggest that the Cooper’s ligaments attached to the pectoralis major muscle play a pivotal role in providing support. Conversely, C bears the least amount of stress at only 4.0 kPa, which means that the Cooper’s ligaments that are in contact with the glandular tissues provide less support.

#### Biomechanical analysis of pectoralis major muscle

**Fig. 10:**
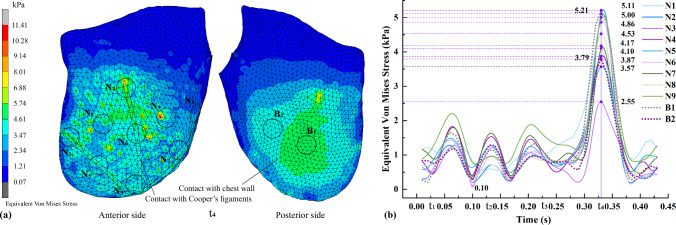
Equivalent von Mises stress of pectoralis major muscle. **a** stress nephogram, and **b** different areas and their average values

Figure 10a shows the equivalent von Mises stress nephogram of the pectoralis major muscle at t_4_. The contact between the surfaces of the pectoralis major muscle and Cooper’s ligaments is outlined with dotted lines. The circular areas (N_1_–N_9_) and (B_1_, B_2_) are on the anterior and posterior sides of the pectoralis major muscle, respectively. Their mean values are plotted in Fig. 10b, with t_4_ still indicative of the moment of peak stress.

Note that the areas of the pectoralis major muscle that are in contact with the Cooper’s ligaments are subjected to considerably more stress, which ranges between 3.47 and 10.28 kPa, as shown in Fig. 10a. This is also reflected in Fig. 10b. On the anterior side, the mean stress of N_1_ and N_2_ is relatively high, which reaches 5.00 and 5.11 kPa, respectively. In contrast, on the posterior side, the mean stress of B_1_ is also the highest at 5.21 kPa, which indicates that the centre of the posterior surface of the pectoralis major muscle bears most of the stress from breast motion.

#### Biomechanical analysis of glandular tissues

**Fig. 11:**
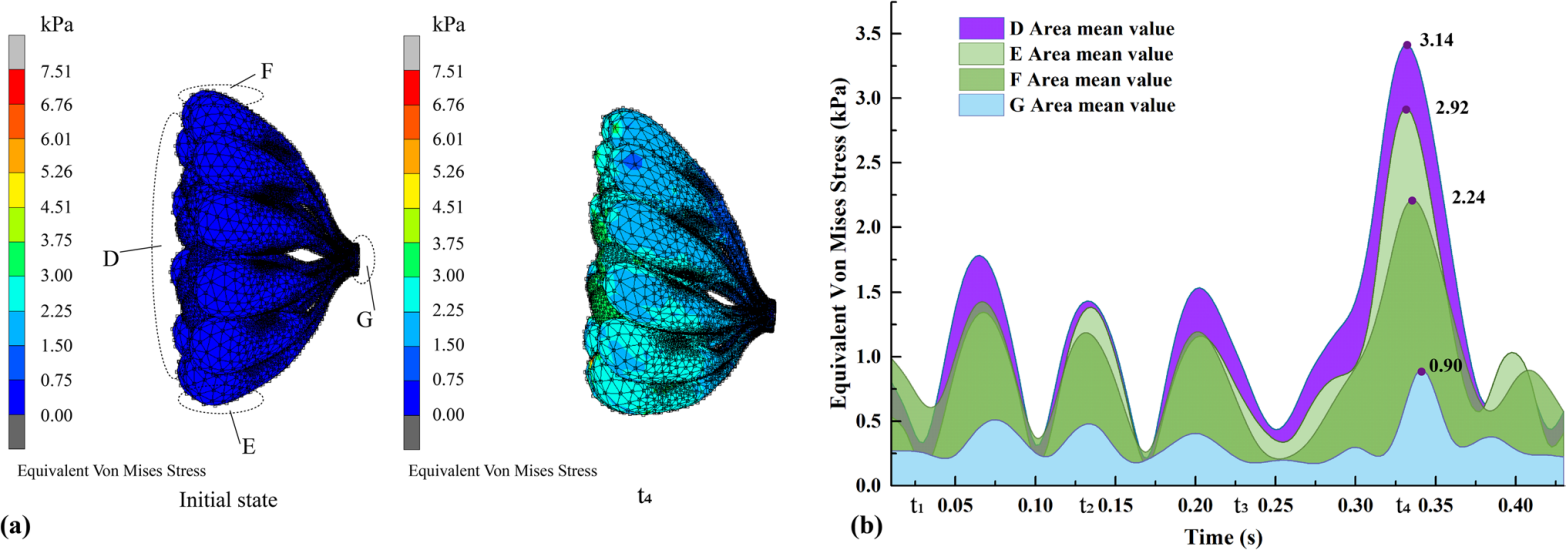
Equivalent von Mises stress of glandular tissues: **a** stress nephogram, and **b** average values of four areas of concern

Figure 11a shows the equivalent von Mises stress nephograms of the glandular tissues in the initial state and t_4_. Areas D, E, F, and G correspond to the rear, bottom side, top, and front of the glandular tissues, respectively. The average stress values for these areas with time were determined. Notable deformation and stress are observed in D and E, compared to the original shape of the glandular tissues. The highest equivalent von Mises stress for D and E is found at t_4_, which reaches 3.41 and 2.92 kPa respectively. Conversely, the stress observed in G is minimal, and does not exceed 0.9 kPa, which suggests that there is very little stress in the sinus area.

#### Biomechanical analysis of adipose tissues

**Fig. 12:**
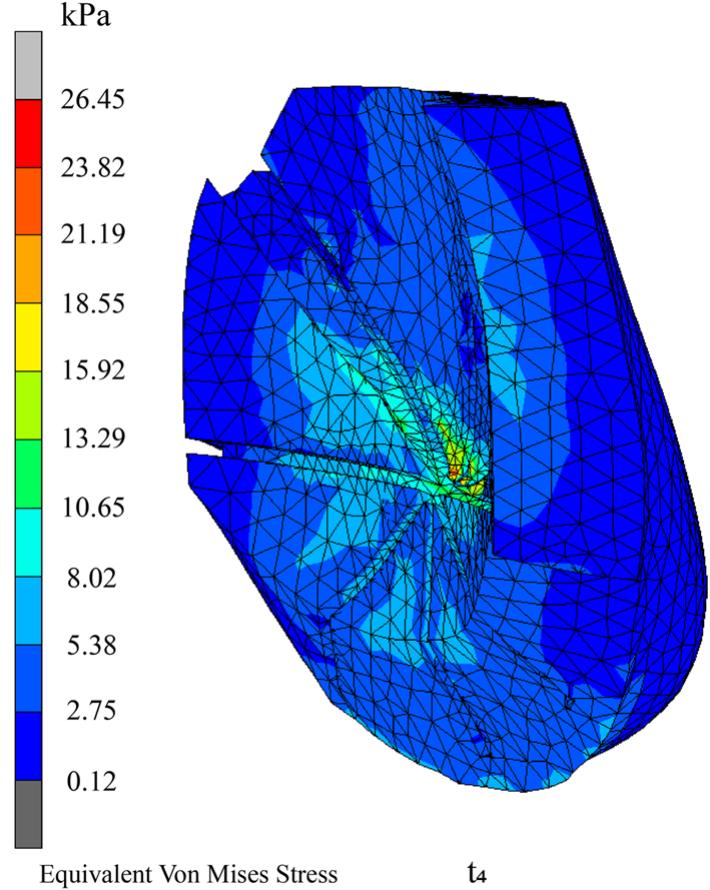
Equivalent von Mises stress of adipose tissues

A much higher equivalent von Mises stress, which ranges from 8.02 to 15.92 kPa, can be observed in the adipose tissues located near the glandular tissues and Cooper’s ligaments, as shown in Fig. 12. This increase in stress is because the glandular tissues and Cooper’s ligaments are harder than the adipose tissues, and hence, interacts with the adipose tissues during movement.

#### Support performance of breast components

The simulation results in this study show that the entire upper and posterior sides of the 3D Cooper’s ligaments, which are attached to the pectoralis major muscle, provide the main support to the breasts. This is evidenced by the relatively high equivalent von Mises stress experienced by the posterior side of the Cooper’s ligaments, which ranges from 4.41 to 30.95 kPa, compared to that of the anterior side of the Cooper’s ligaments, which ranges from 1.68 to 19.17 kPa. The adipose tissues found in proximity to the glandular tissues and Cooper’s ligaments are subjected to higher levels of stress, which ranges between 8.01 and 15.9 kPa. The mean values of the equivalent von Mises stress in the areas of the pectoralis major muscle range from 0.10 to 5.21 kPa. Meanwhile, greater deformation and higher stress are observed on the posterior side and bottom of the glandular tissues, with the stress reaching 3.41 and 2.92 kPa respectively. These results confirm that during breast motion, the Cooper’s ligaments are subjected to the most stress, followed by the adipose tissues, then the pectoralis major muscle, and finally, the glandular tissues, which are subjected to the least amount of stress.

Therefore, during physical movement, the primary supporting structures of the breasts are the Cooper’s ligaments, closely followed by the pectoralis major muscle. These findings are in line with other anatomical research (Gefen and Dilmoney 2007; McGhee and Steele 2020; Riggio et al. 2000). Specifically, Riggio et al. (2000) suggested that these ligaments are considered as the structural framework that supports the breasts. Gefen and Dilmoney (2007) made analytical approximations for the forces that act on the breasts during normal activity and calculated the corresponding internal forces of the breasts supported by the Cooper’s ligaments, pectoral fascia and ribs. Their study concluded that the most highly loaded soft tissue structure in the breasts is the Cooper’s ligament system, either during static standing or while the body is in motion. Meanwhile, Gefen and Dilmoney (2007) also mentioned that the weight of the breasts may induce static or dynamic shear forces, compression or tension, on the pectoralis major muscle. These previous works support the rationality of the findings of this study.

### The effect of different stiffnesses of internal components on breast surface

#### The effect of different stiffnesses of glandular tissue on breast surface

The most important material parameter that affects breast motion is stiffness. This constructed and validated multi-component FE model can be employed to study the impact of varying stiffness levels of internal components on breast surface. Keeping the geometric model unchanged, the stiffness is proportional to Young's modulus (Zhu et al. 2003). In this study, Young's modulus parameter was used to adjust the material stiffness. The Young's modulus of Glandular tissue is typically set to 10 kPa (Sturgeon et al. 2016). Since the stiffnesses of the normal glandular tissues is 1 to 6.7 times that of adipose tissues (set to 3.6 kPa in this model) (Azar et al. 2002; McKnight et al. 2002), in this experiment, maintaining the material parameters of the other breast components constant, Young's modulus of the glandular tissue was set to five level with a gradient of 2.5 kPa, as shown in Table 2.

**Table 2:** Different levels of Young's modulus of the glandular tissue

Levels	1	2	3	4	5
Young's modulus *E* (kPa)	5	7.5	10	12.5	15

**Fig. 13:**
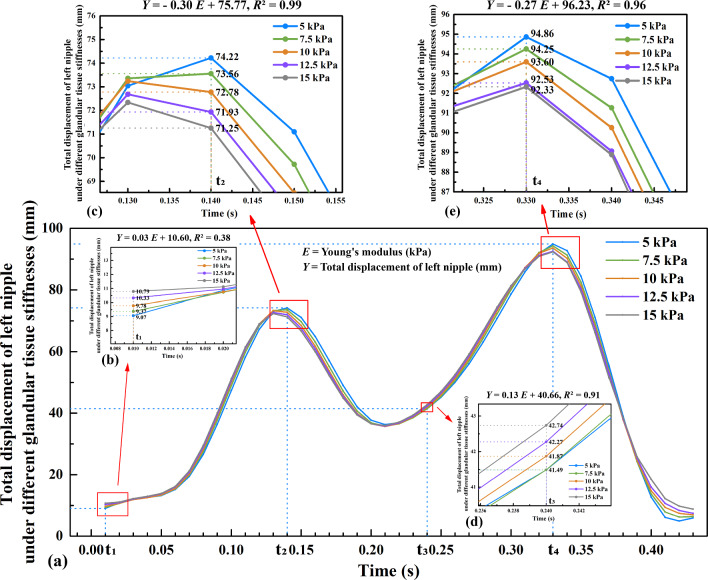
Total displacement of left nipple under different glandular tissue stiffnesses: **a** half a gait cycle, **b** t_1_ moment, **c** t_2_ moment, **d** t_3_ moment, and **e** t_4_ moment

The left nipple was taken as a characteristic point describing the motion of the breast surface. In half a gait cycle, the total displacement of the left nipple under different stiffnesses levels of the glandular tissue can be seen in Fig. 13.

The relationship between Young's modulus *E* and displacement *Y* was linearly fitted. At t_2_ and t_4_, a negative correlation between displacement and Young's modulus was observed, with correlation coefficients of − 0.30 and − 0.27, respectively (see Fig. 13c, e). This indicates that as the stiffness of the glandular tissue increases, the displacement of the left nipple decreases. For instance, at t_2_, a 2.5 kPa increase in Young's modulus led to a decrease in left nipple displacement of approximately 0.75 mm. Similarly, at t_4_, a 2.5 kPa increase in Young's modulus resulted in a decrease in left nipple displacement of about 0.68 mm. This is because higher stiffness results in greater resistance to motion, leading to smaller displacements. At t_1_ and t_3_, a positive correlation between displacement and Young's modulus *E* was found (see Fig. 13b, d), although the correlation coefficients were relatively small, measuring 0.03 and 0.13, respectively.

#### The effect of different stiffnesses of Cooper’s ligaments on breast surface

In this experiment, keeping the material parameters of the other breast components constant, the Young's modulus of the Cooper’s ligament was set to five levels with a gradient of 20 kPa, as presented in Table 3.

**Table 3:** Different levels of Young's modulus of the Cooper’s ligaments

Levels	1	2	3	4	5
Young's modulus *E* (kPa)	80	100	120	140	160

**Fig. 14:**
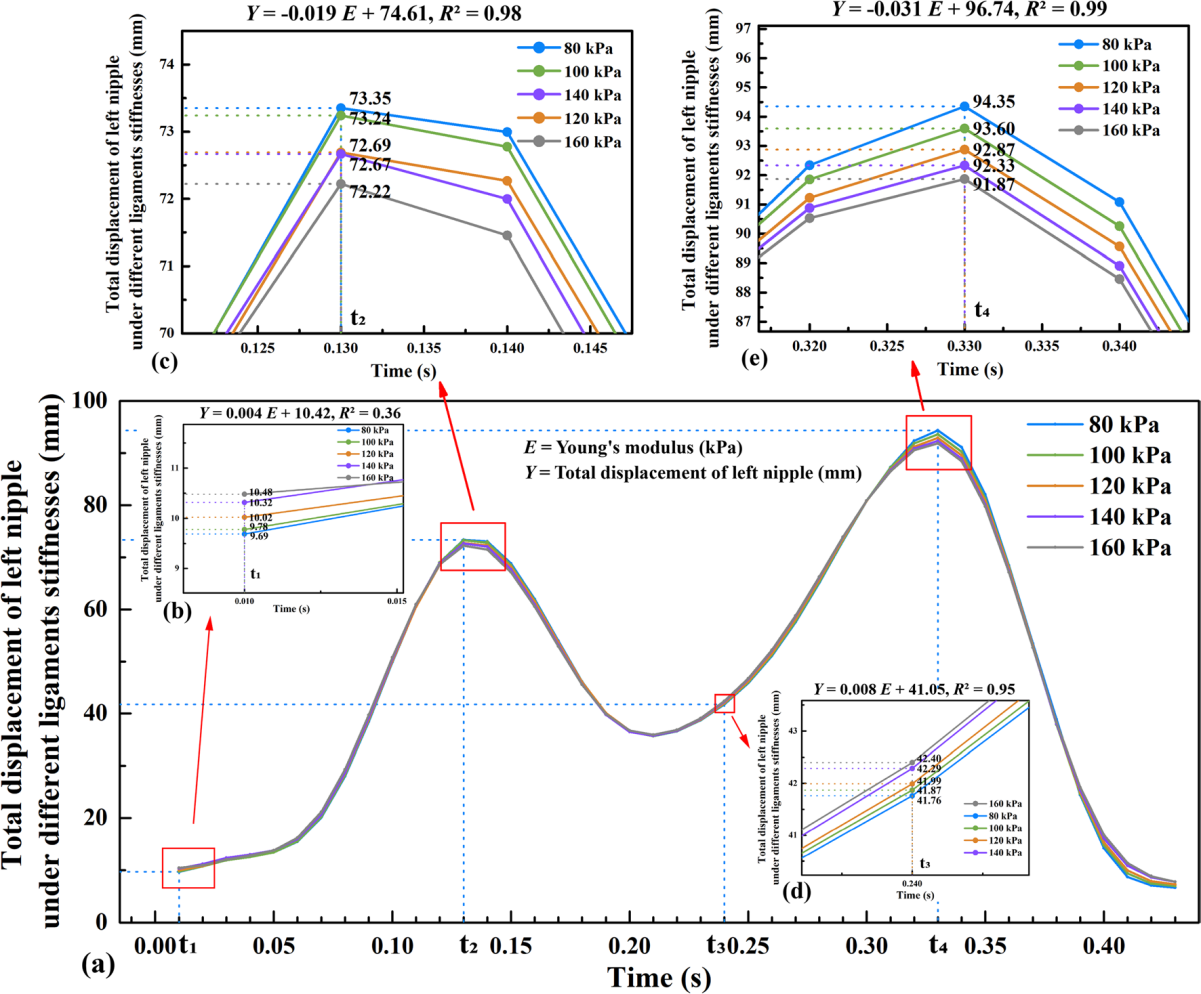
Total displacement of left nipple under different ligaments tissue stiffnesses: **a** half a gait cycle, **b** t_1_ moment, **c** t_2_ moment, **d** t_3_ moment, and **e** t_4_ moment

In half a gait cycle, the total displacement of the left nipple under different stiffnesses levels of the Cooper’s ligaments can be seen in Fig. 14a.

At t_2_ and t_4_, it is found that the displacement is negatively correlated with Young's modulus, and the correlation coefficients are − 0.019 and − 0.031, respectively (see Fig. 14c, e). This shows that the greater the stiffness of the Cooper ligament, the smaller the displacement of the left nipple. For example, at t_2_, for every 20 kPa increase in Young's modulus, the left nipple displacement decreases by approximately 0.38 mm. Similarly, at t_4_, a 20 kPa increase in Young's modulus leads to a decrease in left nipple displacement of 0.62 mm. On the other hand, at t_1_ and t_3_, it is found that the displacement is positively correlated with Young's modulus (see Fig. 14b, d), although the displacement differences in Fig. 14a are very small.

#### Similarity and difference between the influence of glandular tissue and Cooper’s ligaments

Based on the above analysis, the influence of glandular tissue and Cooper's ligament tends to be consistent at the time of maximum displacement during running. The greater the stiffness of the material, the smaller the nipple displacement on the breast surface, showing a negative correlation. This can be attributed to the biomechanical response of breast tissue under dynamic loading. When the Young's modulus of breast tissue, such as glandular tissue or Cooper's ligaments, increases, it indicates higher stiffness or resistance to deformation. In this case, the breast tissue is less able to stretch or deform in response to the force applied. Therefore, when the Young's modulus is higher, the displacement of the left nipple decreases. Pervious study (Samani and Plewes 2004) also mentioned that due to low tissue stiffness and lack of physical constraints, tissue deformation may be very large.

Comparing the influence of glandular tissue and Cooper’s ligaments, it found that under the gradient of Young's modulus, the change in stiffness of the glandular tissue has a slightly greater influence on the surface of the breast compared to the Cooper's ligaments. This can be attributed to the fact that the volume of glandular tissue is typically larger than that of the Cooper’s ligaments (Gaskin et al. 2020a, b; McGhee and Steele 2020; Rehnke et al. 2018). Consequently, altering the stiffness of the glandular tissue material has a more significant effect on the behaviour of the breast surface.

## Conclusion

A nonlinear multi-component dynamic FE model has been established in this study to simulate breast biomechanical behaviour and quantify stiffness impact during running. Through 4D scanning technology, a model of the body with the breast is used as the geometric model, which is constructed as a breast model with different components that include the skin, a layer of soft tissues, the pectoralis major muscle, adipose tissues, Cooper’s ligaments and glandular tissues. Both the traditional key point-to-point method and the latest surface-to-surface method are used to validate the FEM-simulated results, which are shown to be in agreement with the experimental results. By simulating the motion of the different components of the breasts, this study conducts displacement, biomechanical analyses, and the stiffness impact of the components of the internal structure of the breasts. The result reveal that at the moment of maximum displacement, the displacement of the different breast components is proportional to their distance from the rigid chest wall. The findings also reveal the relationship for stress with the different breast components. During breast motion, the Cooper’s ligaments provide the primary support, followed by the pectoralis major muscle. Moreover, specific positions where the breast components are subjected to stress are identified. In comparison to the Cooper's ligaments, changes in the stiffness of the glandular tissue have a slightly more pronounced impact on the breast surface.

The results of this study have several implications. First, the results enhance understanding of breast anatomy and biomechanical behaviour. Second, this breast biomechanical modelling method quantifies the internal structure of the breast, enabling quick application to different age groups or specific patient groups for simulating internal biomechanical behaviour during motion. In terms of applications, this study identifies stress positions of breast components, such as the upper and posterior sides of Cooper's ligaments and posterior side and bottom of glandular tissues. These findings are valuable for the sports bra industry, highlighting the need for support in critical areas while avoiding compression of major supportive components like the upper part of Cooper's ligament. Results enable anatomically engineered sports bras with enhanced support and protection. Moreover, understanding the stress distribution in different breast components aids in designing prostheses and support devices that align with anatomical and functional requirements for breast cancer patients undergoing mastectomy and reconstruction.

Finally, one limitation of this study is that a theoretical model of the breast customised to a single individual was used. Breast tissue comes in varying sizes, compositions, and stiffnesses that are not taken into account in this study. In future work, we aim to include more diverse breast models of different breast shapes and age groups to optimise the model further. Additionally, there is a discrepancy between the ligament model of this study (resembling a cable) and the authentic anatomical configuration of breast ligaments (exhibiting a network-like structure). This study primarily investigates the supportive effects of ligaments in the vertical direction, so the relevant conclusions also need to be carefully adopted. Future studies could explore using a mesh structure to model breast ligaments to narrow this gap.

## Data Availability

The data that support the findings within this study are available from the corresponding author upon a reasonable request.
